# Experimental Researches on Influence of Bitter Herbs on the Function of the Sound and Diseased Stomach

**Published:** 1890-03

**Authors:** 


### EXPERIMENTAL RESEARCHES ON INFLUENCE OF BITTER HERBS ON THE FUNCTION OF THE SOUND AND DISEASED STOMACH.

N. Reichmann (Zeitschrift f. klin. Med., Bd. xiv, Heft 1 and 2; Ctrlbl. f. klin. Med., No. 49, 1889) has made a number of experiments on man in reference to this subject. His results may be summed up as follows:

1. The action of the different bitter remedies is about equal (herba centaurt, folia trifolii fibrini, radix gentianæ, lignum quassiæ, herba absinthii).

2. In every fasting stomach, whether with normal secretion or attended with diminished or increased gastric secretion, the introduction of a bitter infusion is followed by a greater secreting activity than when simple distilled water is used.

3. When the bitter infusion is taken on an empty stomach, it stimulates the gastric secretive apparatus for some time after it has disappeared from the stomach.

4. Bitter infusions given at the same time food is taken, interfere with the digestion of the latter; they seem also to interfere with the mechanical activity of the stomach.

5. The employment of bitter infusions for several weeks produces no functional changes in the healthy or diseased stomach.

Of practical importance is paragraph No. 3, which teaches us that bitter infusions should be given only when the secretory of the stomach is defective. In these cases the remedy should be taken half an hour before partaking of food.—*New York Medical Journal.*

